# Why women choose compounded bioidentical hormone therapy: lessons from a qualitative study of menopausal decision-making

**DOI:** 10.1186/s12905-017-0449-0

**Published:** 2017-10-02

**Authors:** Jennifer Jo Thompson, Cheryl Ritenbaugh, Mark Nichter

**Affiliations:** 1 Department of Crop and Soil Sciences, University of Georgia, 3111 Miller Plant Sciences, Athens, GA 30602 USA; 2 Department of Family and Community Medicine, University of Arizona, 1450 N. Cherry Ave, Tucson, AZ 85719 USA; 3 School of Anthropology, University of Arizona, 1009 E. South Campus Drive, Tucson, AZ 85721 USA

**Keywords:** Menopause, Hormone therapy, Bioidentical hormones, Compounded hormones, Shared decision-making, Qualitative research

## Abstract

**Background:**

In recent years, compounded bioidentical hormone therapy (CBHT) has emerged as a popular alternative to manufactured, FDA approved hormone therapy (HT)—despite concerns within the medical community and the availability of new FDA approved “bioidentical” products. This study aims to characterize the motivations for using CBHT in a U.S. sample of ordinary midlife women.

**Methods:**

We analyze data collected from 21 current and former users of CBHT who participated in a larger qualitative study of menopausal decision-making among U.S. women. Interviews and focus groups were audio-recorded, transcribed verbatim, and analyzed thematically using an iterative inductive and deductive process.

**Results:**

Although women’s individual motivations varied, two overarching themes emerged: “push motivations” that drove women away from conventional HT and from alternative therapies, and “pull motivations” that attracted women to CBHT. Push motivations focused on (1) fear and uncertainty about the *safety* of conventional HT, (2) an *aversion* to conjugated estrogens in particular, and (3) and overarching *distrust* of a medical system perceived as dismissive of their concerns and overly reliant on pharmaceuticals. Participants also voiced dissatisfaction with the effectiveness of herbal and soy supplements. Participants were attracted to CBHT because they perceive it to be (1) *effective* in managing menopausal symptoms*,* (2) *safer* than conventional HT*,* (3) *tailored* to their individual bodies and needs, and (4) accompanied by enhanced clinical care and attention.

**Conclusions:**

This study finds that women draw upon a range of “push” and “pull” motivations in their decision to use CBHT. Importantly, we find that women are not only seeking alternatives to conventional pharmaceuticals, but alternatives to conventional care where their menopausal experience is solicited, their treatment goals are heard, and they are engaged as agents in managing their own menopause. The significance of this finding goes beyond understanding why women choose CBHT. Women making menopause treatment decisions of all kinds would benefit from greater shared decision-making in the clinical context in which they are explicitly invited to share their experiences, priorities, and preferences. This would also provide an opportunity for clinicians to discuss the pros and cons of conventional HT, CBHT, and other approaches to managing menopause.

## Background

Since the discovery of sex hormones in the late nineteenth century, women’s relationship with hormone therapy (HT) for menopause has endured a series of paradigm shifts [1, 2]. It has been framed as a panacea for menopausal symptoms and the diseases of aging [3–8], as well as a source of risk [9–11]. Nevertheless, by the end of the twentieth century, HT was touted as the responsible choice for women who sought to protect themselves from chronic diseases as they age [12] and estrogen was the top-selling prescription drug in the U.S. [13]. In this context, the 2002 and 2004 discontinuations of the Women’s Health Initiative (WHI) clinical trials [14, 15] once again up-ended conventional wisdom about HT. In response to highly publicized findings that the risks of HT outweighed the benefits for the prevention of certain chronic diseases, many menopausal women quit HT [16, 17] and sought alternatives for managing the symptoms of menopause. Since then compounded bioidentical hormone therapy (CBHT) emerged and continues to be a popular alternative to manufactured, Food and Drug Administration (FDA)-approved HT [18, 19]—despite concerns voiced within conventional medicine [20–22] and the availability of new lower-dose and “bioidentical” products that carry FDA approval.

### Compounded bioidentical hormone therapy

Compounded hormone therapy is form of bioidentical hormone therapy that is individually formulated for patients by pharmacists. Popularly, the term “bioidentical” refers to prescription hormones that have “the same molecular structure as a hormone that is endogenously produced and circulates in the human bloodstream” ([22] p. 1319). Bioidentical hormone therapy may be manufactured in standard doses by drug companies and sold under brand names such as Vivelle (estradiol) and Prometrium (micronized progesterone). Alternatively, it may be individually formulated for patients by compounding pharmacists as CBHT. CBHT is available in an array of delivery methods (e.g., capsules, patches, creams, sublingual lozenges or “troches”, and vaginal suppositories) and dose strengths, although common compounded formulations include estriol alone, “bi-estrogen” or “bi-est” combinations (estradiol and estriol), or “tri-estrogen” or “tri-est” combinations (estrone, estradiol, and estriol)—as well as progesterone, testosterone, and dehydroepiandrosterone (DHEA) [23, 24]. Unlike conventional HT and manufactured bioidentical hormones, CBHT is *not* regulated and approved by the FDA because it is not a standardized pharmaceutical product; thus, CBHT does not carry the safety warnings mandated by the FDA for estrogen-products after the discontinuation of the Women’s Health Initiative [25].

### Safety and efficacy of CBHT

Data on the safety and efficacy of CBHT is limited. With some exceptions [26], medical and scientific consensus generally holds that bioidentical hormones likely have the same risks and benefits as conventional hormones [23, 24, 27–29]. At the same time, these and other authors raise concerns particular to CBHT—most notably, the potential for exposure to higher doses of estrogen than necessary for symptom control, inadequate doses of progesterone required to protect against endometrial cell proliferation due to inconsistencies in the quality of compounded pharmaceuticals, and the use of serum or salivary hormone testing to determine dosage [22, 28, 30–33]. In general, the medical community recommends FDA-approved HT over CBHT to ensure product quality, and recommends that hormone regimens be clinically tailored to comply with FDA recommendations for using the lowest dose needed to achieve treatment goals [20–22, 34–36].

### Prevalence of CBHT use

Although there is little historical data on the prevalence of CBHT use, it appears that its popularity rose dramatically among women following the halt of the WHI hormone therapy clinical trials, when many women stopped using conventional HT and sought alternative approaches for managing menopausal symptoms [13]. In the years that followed, there were also a number of high profile endorsements, including from Oprah Winfrey, who declared, “After one day on bioidentical estrogen, I felt the veil lift” [37].

**Fig. 1:**
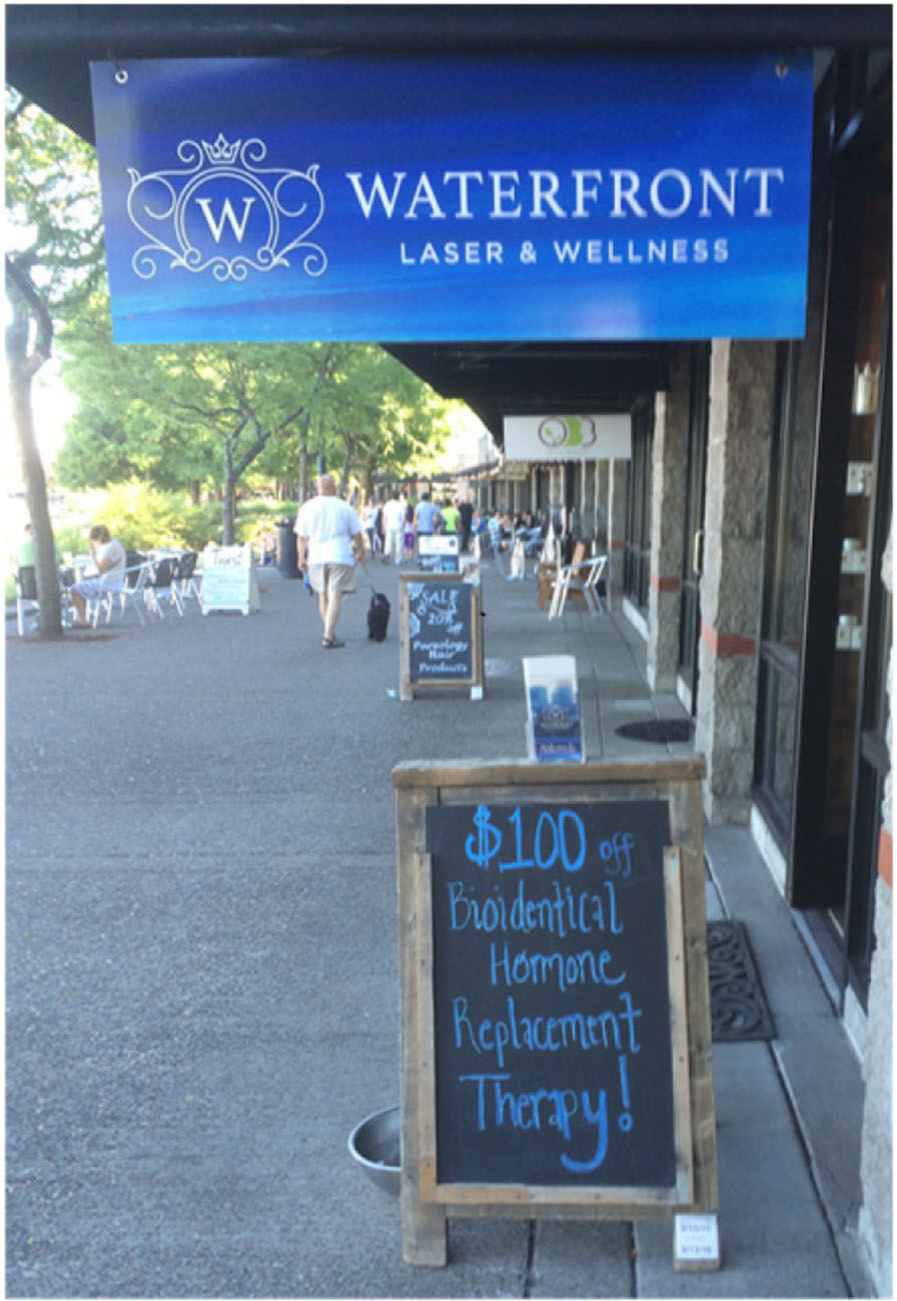
Storefront promotion of CBHT in Portland, Oregon, 2016. (Photo: Jennifer Jo Thompson)

Today CBHT remains hotly debated and in the news [38, 39], and recent data suggests that CBHT use remains high: A recent, national population-weighted survey determined that 35% of U.S. women currently using HT (and 41% of U.S. women aged 40–49 who have ever used HT) are using CBHT [18]; others have similarly estimated that 1 to 2.5 million U.S. women over the age of 40 are currently using CBHT [19]. A survey of U.S. pharmacies estimated 26–33 million annual CBHT prescriptions, totaling $1.3–1.6 billion U.S. dollars—most of this in out-of-pocket spending [40]. Most of these pharmacies projected growth in compounding around 5% - 25% over the next 2 years (Fig. 1). The only national-level, population-based study of the prevalence of CBHT to date comes from Australia, where authors find that 6% of women aged 50–69 have used CBHT and 2% are current users [41].

### Understanding CBHT use

Given the continued popularity of CBHT among women despite efforts on the part of the pharmaceutical industry to provide new forms of manufactured BHT, understanding *why* women choose CBHT is essential. Yet, few studies have actually examined women’s experiences with CBHT or their rationales for using it, with most focusing on what women may not know or understand about CBHT. For example, a 2005–2006 survey of 184 patients attending an academic clinic for menopausal care, found that 77% of current or ever users of CBHT believed it to be “safer” than conventional HT [42]. More recently, Pinkerton & Santoro [19] found that only 14% of menopausal women knew that CBHT lacks FDA approval. Countless other papers and position statements take women’s lack of knowledge or misunderstanding about CBHT as a given, and assert the need to dispel myths and educate women on the facts around CBHT [20, 21, 32, 43]. Although it is common to assume that public skepticism or resistance to mainstream science stems from a lack of knowledge or understanding of the scientific facts [44, 45], there is also increasing recognition that scientific understanding is only one of many domains that people draw upon when making decisions, and providing more information alone is not change attitudes or behaviors [46–49].

Few studies have actually explored women’s rationales for using CBHT. A mini-focus group with four CBHT users in Canada found that women’s decisions to use CBHT were related to the persistence of menopausal symptoms, the side effects of conventional HT, and a personal preference for CBHT [50]. An Australian survey of 366 women using compounded progesterone identified symptom improvement, the perception of compounded progesterone as safe and natural, and the tailoring of treatment as key factors impacting the acceptability of compounded progesterone to women [51]. In interviews with 25 women seeking anti-aging care, all of whom reported using CBHT, Fishman and colleagues found that women pursued anti-aging services for two overarching reasons: first, for treatment of symptoms they attributed to menopause-related hormonal imbalance or decline, and second, out of frustration with conventional medical care they viewed as “unwilling or unable to adequately treat their hormonal imbalance” ([52] p. 83).

### Significance of this research

This work is situated within a larger body of work that aims to understand health care decisions within an increasingly “patient-centered” health care paradigm that encourages shared decision-making between patients and clinicians.^1^ This is especially relevant in a health care environment where information and treatment options—each with their own risks, benefits, and side effects—proliferate. In this context, there is increasing recognition by clinicians that patients’ priorities and preferences matter [53]. A systematic review of patient-reported barriers to and facilitators of shared decision-making identified patients’ *knowledge* (about treatment options as well as their own preferences/goals) and patients’ perceived *power* to participate (e.g., receiving explicit permission from clinician, and having the confidence and self-efficacy to voice their preferences) as key themes influencing patients’ participation in shared decision-making [54]. However, there is also compelling evidence that merely providing patients with more information is not sufficient to support shared decision-making. Rather, “[p]atients need to feel supported so they feel capable of acquiring and understanding knowledge about the available options, and so that they value their personal knowledge contribution” ([54] p. 3017).

The variability of the menopausal experience, the proliferation of information and treatment options, and the continued debate in the medical community about the risks and benefits of hormone therapy makes menopause management an ideal case for investigating the complexities of patients’ participation in decision-making. This study seeks to identify and understand the motivations for choosing and using CBHT in a U.S. sample of ordinary midlife women. Analyzing interview and focus group data collected with current and former users of CBHT, this paper identifies motivations that drive women away from conventional approaches to managing menopause, and those that attract women to CBHT in particular. These motivations reveal the complexities of health decision-making, in which individuals view scientific information through lenses of bodily and social experience. Most importantly, they reveal the value of clinical care in which women feel that their menopausal experience is solicited, their treatment goals are heard, and they are engaged as agents in managing their own menopause. For the vast majority of women, paying out-of-pocket for CBHT is not an option—but understanding why some women make this choice can improve menopausal care for all women.

## Methods

Qualitative research serves as an important complement to experimental and observational quantitative research in the health sciences: it lays the groundwork for good quantitative research; it strengthens interpretation of quantitative data; and it allows for the investigation of unfolding social and organizational processes that are difficult to study in any other way [55]. In particular, qualitative research allows researchers to “understand social phenomenon in natural (rather than experimental) settings, giving due emphasis to the meaning, experiences, and views of all participants” ([55] p. 43).

The data reported here were collected as part of a larger ethnographic study of menopausal decision-making among women living in the southwestern US. Ethnographic research, rooted in the discipline of anthropology, utilizes a broad set of research methods—including participant observation, informal conversations, focus groups, and semi-structured and open-ended interviewing—to investigate individual experience and collective behavior within its social context [56–58]. Like phenomenology, ethnographic research examines the lived-experience (individuals’ direct and subjective experiences) and, as with grounded theory, analysis focuses on developing explanatory theories from the data, rather than imposing theoretical frameworks from above [59].

This paper reports on interview and focus group data from 21 women who were current (11) or former (10) users of CBHT. Our data were collected in 2007–2008, approximately five years following the halt of the Women’s Health Initiative. A decade hence, the WHI continues to have ripple effects in women’s menopause management decisions. Although organizations like the North American Menopause Society and the UK’s National Institute for Health Care Excellence have sought to encourage women and clinicians to (re-)consider HT as an appropriate treatment for menopausal symptoms [36, 60], media and information sources continue to index the WHI as a salient reference point in women’s experience—reflecting women’s (and doctors’) continued uncertainty about risk related to menopause management e.g., [38, 61–64]. Further, although several professional societies have issued statements highlighting their concerns and discouraging the use of CBHT [20–22], data (as we review above) indicates that women are still choosing CBHT in large numbers. Yet there remains a lack of qualitative research illuminating *why* women make this decision. Finally, women do not make health care decisions in a vacuum; rather, they solicit advice from others who have been through a similar experience [65, 66]. Women entering menopause today are soliciting advice from those who entered menopause ahead of them. These are the women we interviewed. Thus, the experiences and rationales we report are the same experiences and rationales older women are sharing with their younger sisters and friends entering menopause today. In short, the environment in which women are making menopause decisions has not changed substantially enough to make these data obsolete. Rather, they make an important contribution to understanding women’s motivations and rationales for choosing CBHT.

Participants for the overall ethnographic study were recruited through a variety of women’s health clinics, community advertisements, and personal referrals to represent a range of menopausal symptom experiences and strategies for managing menopause, as well as to ensure racial/ethnic diversity and sexual orientation.^2^ To facilitate a comprehensive study of women’s menopausal experience and management strategies [67], we did not recruit or exclude on the basis of menopausal status or treatment status; rather, any English-speaking woman who self-identified as going through menopause was eligible to participate.

Semi-structured interviews were designed to elicit in-depth information about women’s personal experiences, perspectives, and strategies for managing menopause, including information-gathering and treatment-seeking [68, 69]. Focus groups explored similar topics in a group setting [70]. Both interviews and focus groups lasted 90–120 min. They were audio-recorded and transcribed verbatim, with the exception of one focus group when equipment failed. Detailed field notes were taken during all segments of data collection.

Transcripts and field notes from all phases of data collection were imported into ATLAS.ti, qualitative data management software, for coding and thematic analysis. At its core, thematic analysis aims to identify and make sense of important patterns that emerge in the data [71, 72]. Data were analyzed using an iterative process. Guided by a preliminary set of themes from the literature and research questions, JJT conducted an inductive analysis of field notes from interviews and focus groups to generate a list of structural and descriptive codes emerging from the data. This process continued until conceptual categories were saturated—that is, until no new codes emerged and the definitions had stabilized [73, 74]. In consultation with co-authors, JJT identified initial themes found in the data and refined the exhaustive list into a set of 46 codes with clear conceptual boundaries that reflected our central research questions and emergent issues (e.g., description of menopause; information assessment; healthcare interaction; treatment perceptions; social support). JJT used this code list to analyze transcripts of all ethnographic interviews and focus groups, in ongoing consultation with co-authors [75, 76].

In this paper, we focus on a subset of interviews and focus group data from the 21 participants who were current or former users of CBHT. JJT re-analyzed these interviews to identify women’s motivations for using CBHT. This entailed reviewing the interviews in their entirety, generating a list of codes based on the values, attitudes, and beliefs that influenced participants decisions to use CBHT, organizing these codes to identify themes related to women’s motivations, and re-coding each of the 21 interviews/focus groups for these themes [71, 77]. Findings were discussed with co-authors to consensus.

The University of Arizona’s Institutional Review Board approved this research, and all participants provided written informed consent.

## Results

Twenty-one women (25.6%), from among this larger study of 82 participants, reported having ever used compounded hormone therapy. Of these, eleven women (52.4%) were currently using CBHT, while ten (47.6%) were former users. While many other participants expressed interest in CBHT, our analysis focuses on the experiences and rationales of the 21 women who had actually used it. Table 1 summarizes the demographic characteristics of women in this subsample. Table 2 provides details about each participant. Compared to the larger study, the women using CBHT in this study were overwhelmingly well-educated and professional women, and they were active participants in their menopause-related information seeking and decision-making. Like the women seeking menopausal treatment in the overall study, those choosing CBHT said that the symptoms of menopause were disrupting their personal or professional lives. CBHT users, in particular, highlighted the value of managing their symptoms for their quality of life and for their ability to function with minimal disruption. It is important to note, however, that many women in the broader sample described experiencing only minor or intermittent menopausal symptoms that did not disrupt their lives in substantial ways.

**Table 1:** Summary demographics of sub-sample of current and former CBHT users

	Current CBHT users (*n* = 11)	Former CBHT users (*n* = 10)
Menopausal Status	9 postmenopausal	6 postmenopausal
	2 perimenopausal	3 perimenopausal
		1 hysterectomy
Age	49–63 (median 54)	39–63 (median 54.5)
Race/Ethnicity	10 white	8 white
	1 African-American	1 African-American
		1 Latina

**Table 2:** Participant Characteristics

	Pseudonym	Race Ethnicity	Occupation (self-report)	Education level	Menopausalstatus	Conventional HT ^a^	Herbal or Dietary Supplements	CBHT Clinician^b^	Type of CBHT^c^	Duration of CBHT (at interview)	Primary Menopause Information Sources	Reason for stopping CBHT
Current CBHT Users	Liz	white	Teacher	Bachelors Degree	Perimenopause	Never	Current	CNM	E & P	2 months	Trusted health care providers. Internet. Health food stores. Books (Christiane Northrup).	n/a
	Francis	white	Therapist	Bachelors Degree	Postmenopause	Never	Current	MD; Nurse Practitioner	E, P, DHEA	Several years	Social and professional networks.	n/a
	Kristina	white	Unemployed	Graduate Degree	Postmenopause	Never	Current	CNM	P	6–7 years	Health care provider. Books (Susun Weed)	n/a
	Deborah	white	Preschool Director	Bachelors Degree	Perimenopause	Never	Never	Naturopath	E & P	2 years	Friends. Trusted health care provider. Books. Health seminars. Media. Health food stores.	n/a
	Bev	white	Certified Nurse Midwife	Graduate Degree	Postmenopause	Never	Prior	Self (CNM)	E & P	6 wks	Media. Medical journals. Friends. Other health care professionals.	n/a
	Dorothy	white	Manager	High School	Postmenopause	Never	Prior	MD	E, P, T, DHEA	6–7 years	Friends. Health food store.	n/a
	Edria	African-American	Retired (researcher)	Graduate Degree	Postmenopause	Never	Prior	MD	E & P	1 year	Medical literature.	n/a
	Sydney	white	Self-employed	Graduate Degree	Postmenopause	Prior	Current	MD	E, P, T	Several years	Internet. Podcasts. Friends. Health care providers.	n/a
	Sandra	white	Substitute Teacher	Graduate Degree	Postmenopause	Prior	Prior	MD (menopause specialist)	T (also using FDA-approved E & P)	6 months	Books. Internet. Friends.	n/a
	Peg	white	Physical Therapist	Bachelors Degree	Postmenopause	Prior	Prior	Naturopath	E & P	12 years	Friends. Books (Northrup). Media.	n/a
	Ann	white	Fitness Instructor	Some College	Postmenopause	Never	Prior	MD (anti-aging specialist)	E, P, T, DHEA	1.5 years	Internet. Friends. Health care providers.	n/a
Former CBHT Users	Kris	white	Self-employed	Graduate Degree	Hysterectomy	Current: FDA-approved BHT	Prior	MD	P	Several years	Internet.	Ineffective; Concern about risk of HT
	Mary	white	PhD researcher	Graduate Degree	Postmenopause	Never	Current	MD	P (also has unfilled Rx for T)	Briefly	Internet. Doctor.	Ineffective; Switched to herbal Progesterone cream.
	Rebecca	white	Retail	Bachelors Degree	Postmenopause	Never	Current	Nurse Practitioner	E & P	1 year	Health food store. Books (Weed).	Negative side effects - stomach, skin, HPV diagnosis
	Joan	white	Behavioral Health Counselor	Graduate Degree	Postmenopause	Never	Never	MD	E & P	1–2 years	Health professionals. Friends. Partner.	Concern about risk of HT
	Sheree	African-American	City Government	Some College	Postmenopause	Never	Never	MD (women’s health specialist)	P	1.5 years	Friends. Critical reading of medical literature.	Symptoms abated; did not refill Rx
	Patsy	white	Retired (nurse)	Graduate Degree	Postmenopause	Never	Never	Internet provider	E & P	5 years	Internet. Books (Northrup).	Concern about risk of HT
	Karen	white	Naturopath	Graduate Degree	Postmenopause	Never	Prior	Self (Naturopath)	E & P	3 wks	Internet (WebMD, Mayo Clinic). Medical journals. Medical conferences. Friends.	Negative side effects - lightheadedness
	Patricia	Latina	Federal Investigator	Some College	Perimenopause	Never	Prior	Naturopath	E & P	4 months	Health care providers. Media.	Negative side effects - weight gain, breast tenderness
	Susan	white	Professor	Graduate Degree	Perimenopause	Never	Prior	MD	E & P	Several months	Internet. Friends. Sister.	Symptoms abated; did not refill Rx
	Kathleen	white	Self-employed	Graduate Degree	Perimenopause	Never	Prior	Naturopath	P	Several months	Internet. Books (Northrup).	Ineffective

^a^HT = hormone therapy

^b^CBHT = compounded bioidentical hormone therapy; CNM = Certified Nurse Midwife; MD = Medical Doctor

^c^E = estrogen; P = progesterone; T = testosterone; DHEA = dehydroepiandrosterone

Although women’s individual motivations varied, several themes emerged across CBHT users that can broadly be categorized into two overarching categories: (1) “push motivations” that drove women away from conventional hormone therapy or from alternative therapies (e.g., herbal and dietary supplements), and (2) “pull motivations” that attracted women to compounded hormone therapy, in particular. Table 3 summarizes the key “push” and “pull” motivations voiced by the current and former CBHT users in this study, and their frequency based on number of participants [78]. In the following section, we discuss each of these themes, and illustrate with exemplar quotations from interviews and focus groups.

**Table 3:** Summary and frequency (based on number of participants) of motivations for using CBHT

Motivations for using CBHT	# (%) of CBHT users
Push away from conventional therapies	
Fear and uncertainty about the safety of HT	17 (80.9%)
Distaste for conjugated estrogens, in particular	10 (47.6%)
Distrust of biomedicine and the pharmaceutical industry	20 (95.2%)
Push away from alternative therapies	
Ineffective symptom management	13 (61.9%)
Pull toward CBHT	
Effective symptom management	16 (76.2%)
Perception that CBHT is “safer” than conventional HT	16 (76.2%)
Desire for individualized treatment	12 (57.1%)
Enhanced clinical experience	13 (61.9%)

### Push away from conventional therapies

To some degree, every one of the 21 current and former CBHT users in this study expressed distrust and frustration with the mainstream medical approach to managing menopause. Many framed their use of CBHT in terms of an express desire to avoid conventional hormonal approaches based on three key themes: (1) fear and uncertainty about the *safety* of conventional HT; (2) a strong *aversion* to conjugated estrogens in particular, and (3) and overarching *distrust* of a medical system that they perceived to be dismissive of their concerns and overly reliant on pharmaceuticals in place of greater clinical attention.

#### (1) Fear and uncertainty about the safety of HT

Seventeen (80.9%) of the 21 current or former CBHT users described their treatment decision within the context of fear or uncertainty about the safety of conventional (manufactured and FDA-approved) hormone therapy. Like those in the overall study, many women choosing CBHT were concerned about the safety of HT, and WHI results confirmed those fears. Susan,^3^ a 56-year-old professor who sought CBHT from her gynecologist for a short period of time when she started experiencing more intense and more frequent hot flashes, put it this way:

Nothing was bad enough to want me to go to take HRT [hormone replacement therapy], or even consider [it]. … I knew several women who were on it. And I know people who are still on it, who are starting on it and taking it. But I never really, even if I had really bad symptoms, I don’t think I would have rushed for HRT. Just because of my thoughts about biomedicine. And especially natural biological processes, I am not sure that we need to change them.

Like other women in this study, Susan suggests that she was never inclined to seek out conventional HT to manage menopausal symptoms, and media coverage of conventional HT reinforced this belief.

Many CBHT users specifically mentioned increased cancer risk as a reason to avoid conventional HT. This concern stemmed from the media attention to increased rates of breast cancer among HT users, the FDA’s “boxed warning” required for estrogen products, and the overall salience of breast cancer as a major health risk for women beginning in midlife. For example, Patricia, a 54-year-old federal employee who used compounded estrogen and progesterone lozenges for about 4 months, explained, “the [symptoms of menopause] for me have not been severe enough to the point where I would consider taking hormone supplements on a regular basis. Because I don’t, I don’t trust the side effects. The risk of the damage I don’t think outweighs the benefit.” When we follow-up on this point, she continues, “I have personally avoided the synthetic hormones because of the side effects for cancer. And sought the bioidentical in as low a strength as I could have compounded.”

#### (2) Distaste for conjugated estrogens, in particular

Although many of the CBHT users in this study categorically ruled out using manufactured HT, nearly half (10 participants, 47.6% of CBHT users) singled out conjugated estrogens (CE) by brand name: Wyeth products Premarin (CE) and Prempro (CE and medroxyprogesterone acetate). In fact, many CBHT users referenced these *equine-sourced* products as way to differentiate conventional, manufactured HT from the CBHT they used. Consider the following focus group conversation between Dorothy, a 61-year-old postmenopausal registered nurse, and Lisa, a 49-year-old manager just entering perimenopause.

DOROTHY: Well, I make a big distinction between the compounding, that’s not made from Premarin, it’s not made from mare urine, versus the pharmaceutical. And it’s very different, very different base, so I’m not afraid of that -- whereas I never wanted to go on the other, and refused for years to go on anything.

LISA: So, what’s the difference between them?

DOROTHY: Well, the base is just entirely different. It has nothing to do with animals whatsoever. […].

[T]he pharmaceutical ones that you would get from a regular gynecologist who just sends you to the regular pharmacy, are made from mare urine and all kinds of different things. And, not just for the conditions of the animals, is one reason I refuse, but also because the side effects are so much more horrendous.

As this exchange illustrates, for some women, CE becomes a symbol of all they perceive to be wrong with conventional HT—from its animal source, which carries both a visceral disgust and concern about animal cruelty, to its questionable safety and side effects. Further, by focusing their concerns about HT on their particular distaste for and avoidance of CE, women are able to make a distinction between conventional HT and the CBHT they use.

#### (3) Distrust of biomedicine and the pharmaceutical industry

Nearly every CBHT user in this study (20 women; 95.2% of CBHT users) expressed frustration and distrust toward biomedicine or the pharmaceutical industry that shaped their overall desire to avoid conventional HT. In particular, CBHT users in this study describe feeling like women had been repeatedly misled by a pharmaceutical industry that promoted HT as the route to longer, healthier, more youthful lives Bev, a 49-year-old certified nurse midwife who prescribes CBHT and uses it herself, put it this way:

It makes me really angry that they’ve pushed hormones for so long. I mean, for 30 years. And they touted it as “you must be young forever.” I think it had a lot to do with the way our culture is biased, maybe more now—much more now—against aging. And then they find out, “Oop! We were wrong!” It just pisses me off. It … confuses and frustrates me when they have conflicting things—three or four articles in a week that say different things. If I can’t sort it out, how can the rest of the world? It’s very frustrating. Get it together. Figure it out.

Although uncertainties are ever-present in medicine, these women express aggravation with a pharmaceutical industry that many feel has tirelessly promoted HT to women despite real concerns about its safety.

Other participants asserted strong critiques of an overly-interventionist medical system, too heavily dependent on pharmaceuticals as a “quick fix.” Embedded in this critique was the perception that many clinicians were too ready to prescribe conventional HT, rather than work more collaboratively with patients to identify a treatment approach that would effectively manage their symptoms in ways aligned with their overall approach to health and wellness. Women described being disappointed by clinicians who they felt were dismissive of their experiences, the particularities of their treatments goals, and their ongoing uncertainty about whether the risks of HT outweighed the clinical benefits. Sandra, a 50-year-old, highly educated immigrant to the U.S., had spent several thousands of dollars out-of-pocket seeking the “right” treatment for her menopausal symptoms. She says, “So, the health professionals, I’m not very happy with they way they’re doing it right now. I mean, all they do [is] give you medications and go, ‘Come back and see how you feel.’” Instead, Sandra says she’s looking for a clinician who considers her as an individual. She continues:

[My doctor], she’s really knowledgeable, but she’s not listening to what I want from her, and I emphasize, you know, what my lifestyle is. She doesn’t consider my lifestyle to what she prescribes to me. … She’s got a certain line of medication, and she prescribes that to every patient. She doesn’t change her therapy for different patients, I think.

Finally, several participants suggested that clinicians’ dismissal of women’s concerns and their reliance on pharmaceuticals should be understood as implicit gender-bias or racial-/ethnic-bias in health care. Peg, a 61-year-old physical therapist, who had been using CBHT for twelve years at the time of our interview, emphasized the relationship between implicit gender bias in medicine and the necessity for self-advocacy, saying:

I try to be as informed as I can, cuz I don’t trust the medical field to be informed... And I don’t trust them to necessarily care—unless I know the doctor personally, and I know that they’re really intelligent, and that they really have integrity. Cuz a lot of them will throw anything at you. And in this culture, they will still do it to women way, way, way more than men. And I work in medicine, and I know that.

Sheree, a 58-year-old working in city government, focused on implicit racial bias and its consequences for the quality of care for women of color. She explained:

The healthcare system creates doctors who have an arrogance about them, and so that arrogance translates into patient care. … [Substandard care] happens all the time, and that has to do with the arrogance. But also being a woman of color, racism is so ingrained, it is so innate, that a lot of times, people don’t even realize that they’re operating from that basis, because it is so much a part of who they are and their frame of reference. It’s just what they do.

The critiques levied by women like Peg and Sheree were not specific to HT, but they signified an overall mistrust of the medical system that has tangible effects on women’s willingness to seek care and trust the recommendations coming through conventional channels.

Although the particular critiques differed, nearly every woman who used CBHT in this study expressed some level of distrust and frustration aimed at clinicians, the broader medical system, or the pharmaceutical industry. Some of this frustration stemmed from ongoing uncertainties about the risk of HT, but they also voiced broader frustrations—about a medical system too quick to dismiss women’s concerns and resort to pharmaceuticals, thus serving up massive profits for the pharmaceutical industry. Together, women’s distrust of the medical system, along with their particular concerns about the safety of conventional HT, and distaste for CE in particular, provide a very strong motivation pushing them away from conventional HT—thus driving them to seek an alternative.

### Push away from alternative therapies

Widely available over the counter, dietary supplements like soy, red clover, and black cohosh are a convenient alternative for women seeking to avoid conventional HT; nevertheless, for the most part, these products have not proven themselves efficacious in clinical trials [79, 80]. Eighty-one percent of the CBHT users (17 of 21) in this study reported trying dietary supplements at some point to manage symptoms they associated with menopause. Many said that specific herbal or soy supplements (with brand names such as Remifemin or Estroven) had been recommended to them by their health care providers, while others reported receiving recommendations from friends or media sources, or reading the labels to find products to fit their needs. Like others who use dietary supplements [81], many women in this study described supplements as more “natural” than pharmaceuticals, and thus perceived them to be safer. Sandra, who tried black cohosh during her search for the right treatment, put it this way, “I tried it. I tried it before I [went] on hormone therapy. Didn’t like it much. I don’t know about the safety of it, but it’s an herb, so very safe. …It didn’t work.”

However, like Sandra, many participants did not find long-term symptom relief from dietary supplements, and this pushed them to seek CBHT as a more effective alternative. For example, when Patricia first began experiencing symptoms early in her perimenopause, she tried a number of over-the-counter herbal supplements on the recommendation of a friend. When we asked her to tell us about that experience, she said, “I didn’t notice any positive effects with them. I didn’t have any negative effects but as far as assisting … the hot flashes during the day and the night sweats, they didn’t help at all.”

Patricia’s response was typical of the CBHT users in this study, many of whom had tried dietary supplements out of a desire to avoid hormones but eventually sought out CBHT as a more effective intervention. Thus herbal and dietary supplements, can begin as a kind of “pull” toward something perceived to be more “natural,” safer, convenient, and less expensive than biomedical and pharmaceutical interventions. However, if they do not provide adequate relief for menopausal symptoms, they become their own “push” motivation—away from an ineffective treatment and toward a treatment similarly framed as “natural” and often perceived as safer, yet something certainly more effective for symptom management: CBHT.

### Pull toward CBHT

It is within the context of the “push” motivations discussed above that participants framed their attraction to CBHT. In short, for many of the CBHT users in this study, CBHT represented a therapeutic approach that was *both* mainstream and alternative. Four key themes emerged in the way women characterized their attraction to CBHT: (1) it is *effective* in managing their menopausal symptoms*,* (2) they *perceive it to be “safer”* than conventional HT*,* (3) it is *tailored* to their individual bodies and needs, and (4) it was accompanied by enhanced clinical care and attention.

#### (1) Effective symptom management

A key draw for CBHT users was simply that it worked. As a hormonal approach, most women found that CBHT was effective in managing their menopausal symptoms. Although a large number of women in the overall research project were able to weather menopausal symptoms without hormones, other women—including those using CBHT—found their symptoms disruptive enough that they actively sought remedy. And for those who wanted to avoid conventional HT, finding a way to effectively manage disruptive symptoms was no easy task. Over three-quarters of the CBHT users in this study (16 out of 21; 76.2%) emphasized that one of their primary reasons for using CBHT was that was effective in managing the symptoms they associated with menopause, where lifestyle approaches, herbal remedies, and sometimes other pharmaceuticals fell short.

For example, in an effort to manage the symptoms she associates with menopause, including hot flashes, short-term memory loss, and insomnia, Peg tried a number of over-the-counter products, including hormonal creams, Black Cohosh dietary supplements, and soy products. She also tried CE—despite a strong aversion to the very idea. Both the soy and the conventional HT made her sick, and nothing alleviated her symptoms. Eventually, Peg found a naturopathic physician who prescribed CBHT. Within a week, Peg felt that her symptoms were manageable: her short-term memory improved, her sleep improved, she felt calmer, and she regained bladder control. She’s been on the same dose ever since—for the last twelve years. Peg says that she’s “afraid to try anything else because *this works*.” At the end of the interview, when we asked what helped her best cope with menopause, Peg says, “the natural hormones!” She continues, “Without them, I think I’d be dead. I mean, I’m not kidding. I really don’t think I’d be alive. I think I’da become so psycho from not sleeping, my brain might’ve exploded, or I might’ve thrown myself in front of a truck, I don’t know.”

Similarly, Deborah, a 53-year-old pre-school teacher in perimenopause, describes her transition to menopause as a “scary time” marked by a number of physical and emotional symptoms, including anxiety, memory loss, insomnia, and night sweats that made her worry that something was “seriously” wrong with her. She says,

I mean, I was depressed for a couple months. I was like, “This isn’t me. This isn’t me. This isn’t who I am. I’ve never been like this.” But once [my naturopathic physician] started me on the [CBHT], the *very next day* I felt better. It was just like the cloud had lifted. It was *amazing*. And I don’t think it was in my head, either. I’d been so, just like, [sharp intake of breath] and I had energy and I was happy.

In short, for women like Peg and Deborah, who are facing severe symptoms they associate with menopause yet wish to avoid conventional HT, CBHT becomes a compelling approach to treatment because, as a form of hormone therapy, it is very effective.

#### (2) Perception that CBHT is ‘safer’ than conventional HT

Over three quarters of the CBHT users in this study (16; 76.2%) described CBHT as safer than conventional HT. The remainder either characterized the safety as equivocal, or did not make any statements about safety. No one characterized CBHT as less safe than conventional HT. Participants based this perception on a couple of key qualities of CBHT: (a) because it is plant-based, it is “natural”; (b) because it is “bioidentical,” it is a more precise chemical match to their bodies’ endogenous hormones; and (c) because it is tailored to their individual needs, they could use the lowest dose possible to achieve their treatment goals.

Ann best illustrates this perception—and how this pull motivation is embedded in the push motivations discussed above. When describing her experience of perimenopause, Ann described experiencing hot flashes, heavy bleeding, and most significantly, insomnia. Because of her work as a fitness instructor, Ann feels a responsibility to keep her body fit as she ages—for her business, but also because she is a role model for her clients who are aging along side her. Nevertheless, she entered perimenopause with a positive attitude, “I figured, ah, it’s not gonna be such a big deal, and it wasn’t until the lack of sleep … because when you don’t sleep, you’re really cranky.” In response, she tried several herbal and soy supplements, which she says “worked for a while, probably.” Finally, she says, “the lack of sleep … that was what prompted me to seek other treatments.” She continues:

I knew I didn’t wanna take chemical hormones, you know, and so some friends of mine were telling me that they were taking the bioidentical hormones, and you know, so I started researching it online, and reading books, … and you know, I know hormones are hormones, but it just seemed to me that if you could take something that was plant-based and more easily recognized by your body, that that would be a safer alternative.

Here, Ann echoes the push motivations discussed above: dissatisfaction with alternative therapies and a desire to avoid conventional HT. She worked with a local physician specializing in “anti-aging” medicine, and takes a compounded regimen of estrogen troches, progesterone pills, and DHEA and testosterone cream. Although she recognizes that “hormones are hormones,” she believes that CBHT might be “a safer alternative” because it is “plant-based and more easily recognized by your body.” When we follow up on this point, Ann indexes the idea that her compounded regimen is safer because it is a closer chemical match to her body’s endogenous hormones. She says, “Well, the way I understand it is that it’s plant based and that it seems to be identical to the hormones that your body produces, or at least that’s the way that your body recognizes it.”

Nevertheless, it is important to point out that Ann—like several of the CBHT users in this study—is not *uncritically confident* about the safety of CBHT. She continues, “like I say, hormones are hormones, and if you’re not supposed to be on hormones for long periods of time, it probably doesn’t matter whether it’s bioidentical versus the chemical, the manmade, whatever.” In the end, however, Ann has stuck with CBHT because it works—saying, “Within a month I was sleeping. You know, no more mood swings, no more hot flashes, no more night sweats.”

#### (3) Desire for individualized treatment

CBHT users in this study were also attracted to a treatment approach that they perceived to be tailored, or individualized, for their bodies and their treatment goals. Twelve (57.1%) women made this point, framing it in several different ways: First, women indexed a kind of biological tailoring in CBHT—both in terms of a chemical match to their bodies’ endogenous hormones and in terms of tailoring hormone dosages based in either blood or saliva tests for hormone levels, or symptom profiles. For example, when we asked Peg whether the naturopathic physician she found was familiar with compounded HT, she responded:

Very familiar with all of this. Very knowledgeable. Did the saliva test. In a week, I had what my body needed—you know, what level it would actually need, so that they didn’t just throw a bulk amount at you. They actually calibrated it for your system, the levels that were in your system or not in your system.

Here, Peg makes a controversial point, one frequently raised in our interviews with women and in the literature on CBHT, about the value of blood or saliva testing for determining CBHT doses. Overall, women in this study held varied perspectives on the value of blood and saliva testing—often reflecting on conflicting ideas picked up through media, or from their clinicians. Susan illustrates this point:

[My gynecologist] said, doing blood work and all that doesn’t work. Because, she said, there’s so much fluctuations, daily fluctuations … [She said,] “I want to start you on the lowest dose and then see if there’s any change. And then go up from there.” And then … I got that feedback from the compounder [that the dose was too low to be effective], and then *there* they have tests that you can buy and send in to some lab. … And so then it was very confusing to know is she right? Are they right? Is there anything such as a bioidentical hormone anyway?

Despite their uncertainties about the value or accuracy of these tests for calibrating hormone dosages, many women were enticed by the *idea* that compounded hormones were tailored to accurately supplement deficits in particular hormones.

Perhaps more importantly, women reported feeling like their CBHT regimen was tailored to meet their individualized treatment goals—that is, to specifically address the symptoms women found most bothersome (e.g., whether women were most bothered by hot flashes vs. low libido might result in a different hormone cocktail). Bev describes this from her dual perspective of certified nurse midwife and CBHT user:

OK, here’s how I do it. Somebody comes to me and tells me that they’re having these symptoms, and I offer them … a range of options, and it depends on whether they’re perimenopausal or postmenopausal, as well. If they decide they wanna go to [a local compounding] pharmacy, as an option, I draw their a detailed [blood] hormone panel, … [then] send you to [the compounding] pharmacy with a referral form, where they take, like, two trees’ worth of paper of a history and a symptom diary, and [the pharmacist] there talks to you for-- I mean she spends a hour and a half with you. And then they decide exactly what your goals are. I mean, not only just, here are my symptoms, and here’s, you know, what my hormone levels are, but, “What are *your* goals?” Which I just find *awesome*. And, like, my goal was never to have a period again. And she said, “Well, if I do this right, you won’t.” I mean, but some people do want to. They like that up and down thing. And they-- she can compound that, too. And they figure it out, and then they check in with you once a month for several months to make sure everything’s good, and if it’s not, then they reformulate, and they give you something a little different. They tweak it until you’re happy with what you have.

We discuss the importance of this kind of clinical care in the section below, but here we highlight the attention to women’s treatment goals and women’s attraction of the idea that CBHT regimens are tailored to meet these goals.

Women also liked that administration method for their CBHT could also be individualized to their preference (e.g., pill, lozenge, cream). Sheree good-naturedly makes this point during a focus group, while also indexing her critique of the quality of mainstream medicine and the biological tailoring of CBHT:

[Most doctors] essentially give all women the same dosage, which I think is like, what, .625 [mg CE]? And, you know, they give us all the same, but what Dr. C was saying is that we’re not the same. So, by looking at our hormone levels, that gives you an idea of what it is that you are deficient in, and so, the compounding formula is specific to your hormonal levels. So, I was over there taking those blackberry and cream troches-- *[group laughter]*… Yeah, yeah, they do, they make ‘em taste good.

Although the delivery is clearly less important than the safety and efficacy of treatment, it demonstrates that women feel like CBHT (in contrast to conventional HT, which they negatively perceive to be uniform) is finely tuned—both to their bodies’ needs and to their personal preferences.

#### (4) Enhanced clinical experience

Many of the CBHT users in this study (13; 61.9%) described clinicians and compounding pharmacists who were willing spend significant time establishing and building trust with women, listening to them as they describe their symptoms and often intimate experiences with menopause, counseling women around their treatment options, and enlisting them as partners in treatment decisions. Above, Bev described this kind of enhanced clinical experience in which the compounding pharmacist takes significant time to talk with women about their menopausal experiences, work with them to identify treatment goals and preferences, and provide follow-up care to ensure that women are satisfied with their CBHT regimens.

Similarly, Liz, a 51-year-old high school teacher, describes the central roles played by her clinicians and compounding pharmacist in assuaging her concerns about hormones and addressing the symptoms she associates with menopause. Liz described her symptoms as a vicious circle of hot flashes, night sweats, insomnia, and anxiety, saying, “It was so awful, it was insane.” To help break the cycle, Liz tried a series of over-the-counter products, including vitamins, herbal supplements, and progesterone cream; she eventually sought treatment from her midwife and primary care provider (PCP) from whom she requested sleeping pills. When we asked her why she requested sleeping pills rather than something to stop the hot flashes, Liz indexed a common push motivation, saying she was avoiding estrogen because “I’m terrified of getting cancer. … My grandmother died of cancer, and my sister-in-law died of breast cancer. My mom had [cancer and] a mastectomy. … It’s a horrible way to die and I don’t want to do anything that would be conducive to inviting that dysfunction to my own body.”

After a consultation with a compounding pharmacist, Liz refused estrogen but decided to try compounded bioidentical progesterone. Nevertheless, when we asked Liz if there is anything she is reserving in case the symptoms become unbearable again, she indexes another push motivation, stressing that “horse estrogen” is completely off the table but that she might be persuaded to try plant-based estrogen. Liz says that she prefers a “holistic kind of approach” to taking a pill. In the end, however, it is the clinical experience (in this case, with several providers) that matters. Liz says, “I guess it really basically comes down to this, I want to be able to describe my experience to somebody who has a vested interest in our relationship continuing, and have them know like anything there is to know and be able to offer that without agenda.”

In a follow-up conversation, Liz reported that she had started using CBHT (estradiol, estriol, and progesterone) soon after our original interview. When we asked her how she decided to use estrogen despite her concerns, she told us that had been “feeling psychotic from nights and nights of not sleeping.” Her PCP told her that “you have to sleep. You cannot function without sleeping.” Liz discussed her reluctance to use estrogen, her family history of cancer, and her overall concerns with her midwife, who explained that compounded estrogen was “different from pharmaceutically-produced hormones.” Like her PCP, her midwife also stressed that Liz needed to get some rest. While she still has some concerns about the safety of using estrogen, Liz says that the time, attention, and care the compounding pharmacist brought to their interaction was “important in feeling like my decision was OK.” The other factor was pragmatic: the CBHT worked immediately. Within two weeks the hot flashes were gone and the sleeping followed.

Central to this point is that women sought clinicians who take time to listen and to develop trust with their patients. Across the broader study, the most satisfied women regardless of treatment type were those that felt that they had a trusting relationship with a clinician who they felt was personally invested in their well-being. This is a lesson for all clinicians.

### Discontinuing CBHT

As of 2004, the FDA has recommended that HT be used at the “lowest effective dose of for the shortest duration to reach treatment goals” [34]. With the notable exception of Peg who says she plans to continue to use CBHT “until the day I die,” the CBHT users in this study view CBHT as a temporary response to symptoms that requires regular re-evaluation. Although they generally perceive the benefits of CBHT to outweigh the risks, they have no interest in extending their exposure beyond what is necessary.

As we discussed above, nearly half (10; 47.6%) of the CBHT users in this study were *former* users—meaning that they had discontinued CBHT use prior to participating in this study. The former users tried CBHT for the same push and pull reasons discussed above. What is different about the former users is that they generally described menopause symptoms in less distressing terms than the current users, and many only used CBHT for a short period when symptoms became more disruptive to their lives. The motivations that women described for discontinuing CBHT were largely same reasons women in the overall study gave for discontinuing conventional HT: the treatment was ineffective or had too many side effects, they were concerned about the risks of HT, or their symptoms abated and did not return.

The most common reasons women gave for discontinuing CBHT were that they were either ineffective or that the side effects of treatment did not outweigh the benefits. For example, having prescribed CBHT for her patients, Karen, a 52-year-old naturopathic physician, tried CBHT when she noticed her mild hot flashes increasing in frequency and intensity. She also started having night sweats. Karen tried a compounded bi-estrogen (estradiol and estriol) and progesterone for only three weeks before discontinuing the therapy due to the side effects she was experiencing. She explained:

The hot flashes disappeared; the light-headedness came. And at first I was just concerned maybe … I wasn’t doing it correctly, or I need to up the dosage, or lower something. I talked to the pharmacist because then I had the spotting and I thought, oh, well maybe this is because I missed a dose and-- But I didn’t like the feeling of light-headedness.

After discontinuing the CBHT, Karen’s light-headedness subsided. At the time of our interview, minor hot flashes had resumed, and she was trying to manage them through dietary changes and stress management techniques.

A few women discontinued CBHT in response to research highlighting the risks of long term HT. Joan, a 56-year-old behavioral health counselor, started using CBHT in 2000 to be proactive about her sexual health and osteopenia; she quit two years later, along with a wave of women who discontinued hormone therapy when the risks were in the news. She explains, “I think that when that study first came out, it was alarming, and I was part of that trend of women that were deciding *don’t do that anymore!”* As we saw with Ann, above, and as Joan illustrates here, although the CBHT users in this study perceive CBHT to be a better choice than conventional HT, they do not view it as wholly different when assessing risk. In light of new information, women reconsidered their CBHT use, just as women were reassessing HT overall.

Finally, several women described passively discontinuing CBHT simply by not refilling their prescription, only to find that their symptoms were bearable without treatment. Sheree said, “So, and I did that [CBHT] probably for about a year, maybe a year and a half, and then … it was time to go back and you know, I didn’t … and you know, there really wasn’t a need for me to go back.” In other words, many women have taken the FDA admonition to use hormones at the lowest dose for the shortest period seriously, and thus it was common for women to report experimenting with reducing hormone dosage or frequency, or stopping altogether to determine whether they could manage symptoms with less. This kind of experimentation, which we saw among women using CBHT as well as those using conventional HT, is similar to the trend we previously identified among dietary supplement users [82], in which individuals become attentive to, and then privilege, their own embodied experience with treatment over professional and clinical prescriptions.

## Discussion

Recent data suggests that over one-third of U.S. women currently using menopausal HT are using CBHT [18]. Given the popularity of CBHT despite concerns within the medical community and the availability of low-dose and FDA-approved “bioidentical” products, understanding *why* women choose CBHT is essential. Yet, few studies have actually examined women’s experiences with CBHT or their rationales for using it. This study aimed to characterize the motivations for using CBHT in a U.S. sample of ordinary midlife women. Analyzing interview and focus group data collected with current and former users of CBHT, we identified motivations that drive women away from conventional approaches to managing menopause, and those that attract women to CBHT in particular.

A cross-cultural meta-synthesis of qualitative and quantitative studies of women’s attitudes toward HT found that women generally have a positive view of the efficacy of HT for treating the symptoms of menopause and improving quality of life, but they have concerns about the potential side effects (especially cancer risk) and about the uncertainty of evidence for HT [83].^4^ These themes are also prominent in the current study. When explaining their decision to use CBHT, our participants referenced ongoing medical uncertainty about the potential risks of HT—in particular, the risk for cancer [84–87]—as a key reason they sought to avoid conventional HT. They also emphasized a strong distaste for and desire to avoid CE (sometimes called conjugated *equine* estrogens), with many identifying these products by brand name and using emotional language to index their equine-source. This may reflect the effectiveness of decades of public awareness campaigns against these products e.g., [88, 89], as well as their continued prominence in the media and medical literature about menopause and HT [38, 84].

Women in this study also voiced frustration with a medical system that they perceive to be dismissive of their concerns and overly reliant on pharmaceuticals in place of clinical attention. They were even more critical of a pharmaceutical industry that they feel has tirelessly promoted HT to generations of women [3, 12, 90, 91] despite legitimate concerns about its safety [1, 9–11, 92]. This should be understood within the broader context of a number of high-profile scandals [93–96] that have undermined public confidence in the pharmaceutical industry [97] and in biomedicine more broadly [98]. Together, women’s distrust of the medical system, along with their concerns about the safety of HT, and distaste for CE in particular, provided a strong push motivation—away from conventional HT toward an alternative.

In contrast, our participants were attracted to CBHT because—as a form of HT—they viewed it as effective for managing the symptoms of menopause and thus for supporting their quality of life. The importance of this point cannot be overstated. It is the efficacy of HT that has allowed it to weather multiple crises of confidence. Clinicians continue to prescribe HT, and women continue to use HT because it *is* the most effective way to manage the vasomotor symptoms of menopause [99, 100]. Thus, for women who vehemently wish to avoid conventional HT despite facing severe symptoms they associate with menopause, CBHT becomes an attractive alternative.

Our research corroborates previous studies indicating that women are attracted to CBHT because they perceive it to be “safer” or more “natural” than conventional HT, and more individualized to their treatment priorities and preferences [42, 51, 52]. Although some clinicians and researchers have posited that CBHT may have some safety advantages over conventional HT [26, 101], several professional medical organizations have raised concerns about the lack of FDA oversight and quality control of compounded products [20–22]. The literature on CBHT similarly rejects both saliva and blood serum testing as inconsistent and ineffective for determining hormone dose, instead recommending that hormone doses should be as low as possible to effectively manage symptoms [20, 22, 99]; nevertheless, women in this study held varied perspectives on the value of blood and saliva testing—often reflecting on conflicting ideas picked up through the media, or from their clinicians. Regardless of where the science lands, we argue that clinicians should take seriously women’s *perceptions* about the safety and individualization of CBHT—not because we view this as a straightforward example of patient misunderstanding driving the need for patient education, but because it speaks volumes about the kinds of treatments women desire, and because these perceptions are influencing women’s real world treatment decisions.

Finally, perhaps the most significant appeal of CBHT may not be the pharmaceutical itself, but the kind of clinical care that surrounds it. Research has demonstrated the interpersonal value of clinical care for patient wellness and satisfaction [102–104]. Many of the CBHT users in this study described clinicians and compounding pharmacists who were willing spend significant time establishing and building trust with women, listening to them as they describe their symptoms and often intimate experiences with menopause, counseling women around their treatment options, and enlisting them as partners in treatment decisions [54, 105]. This is an anomaly in the U.S. health care system, where patients spend an average of 13–16 min of their appointment time with their clinician [106]. Unsurprisingly, the women in this study viewed this enhanced clinical care in a very positive light—perceiving both a personal connection and a personal investment in their wellbeing.

## Conclusions

In recent years, CBHT has emerged as a popular alternative to manufactured, FDA-approved hormone therapy—despite concerns within the medical community and the availability of new FDA-approved “bioidentical” products. This study investigated the motivations for choosing and using CBHT in a U.S. sample of ordinary midlife women. Although women’s individual motivations varied, several themes emerged across CBHT users that can broadly be categorized as “push motivations” driving women away from conventional hormone therapy and “pull motivations” that attracted women to compounded hormone therapy, in particular.

As clinical decision-support tools for menopause symptom management are released [107] and more FDA-approved forms of bioidentical HT become available, *some* of women’s concerns about conventional HT may be allayed, and some of the characteristics women seek may be met with non-hormonal and manufactured bioidentical HT. Yet an important take home message of this study is that women are not only seeking alternatives to conventional pharmaceuticals, but alternatives to conventional care. This study demonstrates that many women chose CBHT because they want a different kind of clinical experience, in which their experiences of menopause are validated and they are listened to, where their treatment objectives are solicited and prioritized, and where they are invited to play an active role in determining their treatment. In short, the clinical context of CBHT appears to explicitly invite women to participate shared decision-making in ways the standard clinical context does not [54, 105].

The significance of this finding goes beyond understanding why women choose CBHT. We argue that women making menopause treatment decisions of all kinds would benefit from a clinical context in which they are explicitly invited to share their experience of menopause, and voice their treatment preferences and priorities. This would also provide an opportunity for clinicians to discuss the pros and cons of conventional HT, CBHT, and other approaches to managing menopause. Certainly, there are often structural barriers (e.g., short appointment slots) that make shared decision-making difficult in the standard clinical context [108]; nevertheless, unless patients receive “explicit permission” to participate in shared decision-making, they often undervalue their own knowledge and preferences and adopt a passive role in the clinical encounter [54]. As such, these lessons are important for all clinicians attending to menopausal issues, and possibly for all clinicians across medicine.

## Abbreviations

CBHT: Compounded bioidentical hormone therapy
CE: Conjugated estrogens
DHEA: Dehydroepiandrosterone
FDA: Food and Drug Administration
HT: Hormone therapy
WHI: Women’s Health Initiative

## Footnotes

1 The U.S. Institute of Medicine identified *patient-centered care* as one of six key aims for improving the U.S. health care system in the twenty-first century [109].

2 Although we did not recruit or exclude on the basis of education or profession, women in this study (like many other studies of menopause) were highly educated and a number worked in health-related fields. Thus, as recruitment ended and we sought to ensure greater racial and ethnic diversity in our study, we purposively recruited professional women of color, so not to conflate minority status and education or socio-economic status.

3 A pseudonym, as are all other participants names throughout.

4 Culturally-specific studies of menopausal women’s attitudes toward HT, such as Jin et al.’s study among Chinese women which finds that although most women had knowledge about menopause few thought menopausal symptoms should be treated and few were aware of HT [110], corroborate cross-cultural variability in attitudes toward menopause and HT [111, 112].
